# Intestinal Epithelial Cell Brush Border Membrane Cl:HCO_3_ Exchanger Regulation by Mast Cells in Chronic Ileitis

**DOI:** 10.3390/ijms252011208

**Published:** 2024-10-18

**Authors:** Raja Singh Paulraj, Sheuli Afroz, Balasubramanian Palaniappan, Usha Murughiyan, Soudamani Singh, Subha Arthur, Uma Sundaram

**Affiliations:** 1 Department of Clinical and Translational Sciences, Joan C. Edwards School of Medicine, Marshall University, Huntington, WV 25701, USApalaniappan@marshall.edu (B.P.); murughiyan@marshall.edu (U.M.);; 2 Department of Internal Medicine, Joan C. Edwards School of Medicine, Marshall University, Huntington, WV 25701, USA

**Keywords:** Cl:HCO_3_ exchange, DRA, PAT1, chronic enteritis, inflammatory bowel disease, IBD

## Abstract

Malabsorption of NaCl is the primary cause of diarrhea in inflammatory bowel disease (IBD). Coupled NaCl absorption occurs via the dual operation of Na:H and Cl:HCO_3_ exchange in the brush border membrane (BBM) of villus cells. Cl:HCO_3_ exchange is mediated by BBM transporters DRA (downregulated in adenoma) and PAT1 (putative anion transporter 1) in the mammalian small intestine. DRA/PAT1-mediated Cl:HCO_3_ exchange was significantly downregulated in the BBM of villus cells in a rabbit model of chronic ileitis, while Na:H exchange was unaffected. The inhibition of Cl:HCO_3_ exchange was restored in the rabbits when treated with a broad-spectrum immunomodulator, i.e. a glucocorticoid, indicating that the downregulation of DRA/PAT1 is likely to be immune-mediated during chronic enteritis. Mucosal mast cells are one type of key immune cells that are known to proliferate and release immune inflammatory mediators, thus playing a significant role in the pathogenesis of IBD. However, how mast cells may regulate DRA- and PAT1-mediated Cl:HCO_3_ exchange in a rabbit model of chronic ileitis is unknown. In this study, treatment of rabbits with chronic intestinal inflammation with the mast cell stabilizer ketotifen did not affect the mucosal architecture of the inflamed intestine. However, ketotifen treatment reversed the inhibition of Cl:HCO_3_ activity in the BBM of villus cells. This restoration of Cl:HCO_3_ activity to normal levels by ketotifen was found to be secondary to restoring the affinity of the exchangers for its substrate chloride. This observation was consistent with molecular studies, where the mRNA and BBM protein expressions of DRA and PAT1 remained unaffected in the villus cells under all experimental conditions. Thus, this study indicates that mast cells mediated the inhibition of coupled NaCl absorption by inhibiting Cl:HCO_3_ exchange in a rabbit model of chronic enteritis.

## 1. Introduction

Inflammatory bowel disease (IBD) broadly describes two clinical conditions (Crohn’s disease and ulcerative colitis) that are characterized by chronic inflammation of the gastrointestinal tract. Though the etiology of IBD is said to be complex and multifactorial, it is known to occur because of an altered immune system, which results in increased production of immune inflammatory mediators leading to disruption of mucosal epithelial function [1,2]. One major immune component that has been implicated in the onset and progression of IBD is the activity of mast cells [3,4,5,6]. Mucosal mast cells, when activated, release a variety of immune mediators such as histamines, serotonins, proteases, lipid mediators (prostaglandins and leukotrienes), cytokines and chemokines, which are known to be responsible for mediating chronic immune response in the gut in IBD. Moreover, the wide range of immune mediators released in the mucosa during IBD suggests that they may affect the absorption of nutrients and electrolytes that are known to be dysregulated in IBD [7,8,9].

The most important function of the human intestine is the absorption of fluid, electrolytes (Na, Cl), and nutrients. Malabsorption of fluid and electrolytes leads to diarrhea while failure to absorb nutrients leads to malnutrition. In the small intestine, the coupled absorption of Na and Cl leads to the absorption of water. Coupled NaCl absorption occurs via the functional coupling of villus cell brush border membrane (BBM) Na:H exchange (NHE3, sodium–hydrogen exchanger 3/SLC9A3) and Cl:HCO_3_ exchange (DRA, downregulated in adenoma/SLC26A3 and PAT1, putative anion transporter-1/SLC26A6) [10]. The most common debilitating symptom of IBD is diarrhea [11,12,13,14]. There is evidence in the literature suggesting the direct role of immune inflammatory mediators on the dysregulation of electrolyte absorption in IBD [15,16]. Pertinent to this study, it was previously demonstrated in a rabbit model of chronic ileal inflammation, resembling human Crohn’s disease, that Cl:HCO_3_ exchange mediated by DRA/PAT1 was significantly downregulated in the small intestinal villus cell BBM, though NHE3-mediated Na:H exchange remained unaffected [17]. Moreover, the downregulation of DRA/PAT1 was found to be secondary to altered affinity of the exchanger for chloride without an alteration in the exchanger numbers in the BBM. Furthermore, treatment of rabbits with chronic enteritis with a broad-spectrum immune modulator, such as a glucocorticoid, reversed the inhibition of Cl:HCO_3_ exchange by reversing the diminished affinity of the exchangers for chloride, indicating the possible role of immune inflammatory mediators in chloride malabsorption in IBD. However, how mast cell activation and degranulation might specifically regulate DRA/PAT1-mediated chloride absorption in a rabbit model of chronic enteritis is not known.

Given this background, this study was performed to determine whether mast cells were involved in the inhibition of small intestinal chloride absorption in a rabbit model of chronic intestinal inflammation. Further, this study also aimed to determine the mast cell-mediated functional and molecular regulation of DRA/PAT1 during chronic intestinal inflammation as seen in IBD.

## 2. Results

### 2.1. Effect of Ketotifen on the Architecture of the Inflamed Intestinal Mucosa

**Figure 1:**
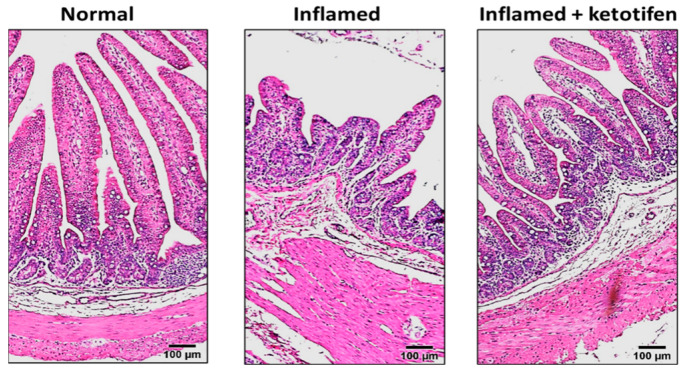
Representative pictures of the cross section of the H&E-stained ileum from normal, inflamed, and inflamed + ketotifen-treated rabbits. The normal rabbit ileum showed long villi, short crypts, and minimal intraepithelial immunocytes. The chronically inflamed ileum showed villus blunting, crypt hypertrophy, and increased intra-epithelial lymphocytes, which are characteristic features of chronically inflamed intestine. Treatment with ketotifen did not alter the characteristic architecture of the inflamed intestine (n = 3). Scale bar 100 µm.

Representative examples of cross sections of the ilea of normal, inflamed, and inflamed + ketotifen stained with hematoxylin and eosin are shown in Figure 1. The control rabbit ileal architecture showed long villi, short crypts, and minimal intraepithelial immunocytes. However, the chronically inflamed ileum of rabbits demonstrated villus blunting, crypt hypertrophy, and increased intra-epithelial lymphocytes, characteristic features of chronic intestinal inflammation in humans. Treatment with ketotifen for two days did not alter the characteristic architecture of the normal or chronically inflamed intestine.

### 2.2. Effect of Ketotifen on Cl:HCO_3_ Exchange in the BBM Vesicles of Ileal Villus Cell

**Figure 2:**
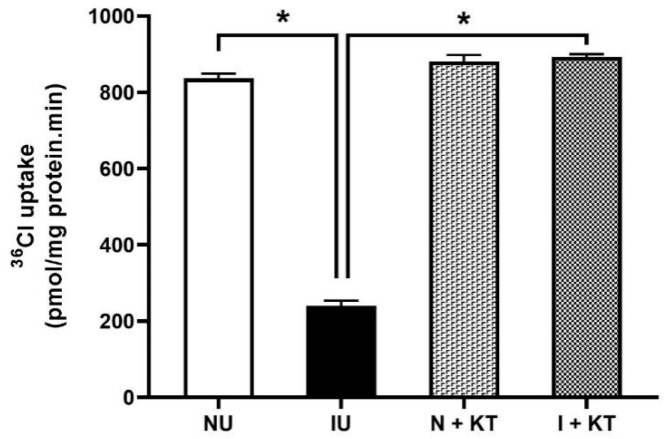
Effect of ketotifen on Cl:HCO_3_ exchange in the BBMVs of ileal villus cells. Cl:HCO_3_ exchange activity (DIDS-sensitive and HCO_3_-dependent ^36^Cl uptake) was significantly decreased in BBMVs of villus cells in chronically inflamed rabbit intestine (N = 4; * *p* < 0.0001). In vivo treatment of ketotifen reversed the inhibition of Cl:HCO_3_ exchange activity in BBMVs of inflamed ileal villus cells. Ketotifen treatment did not alter Cl:HCO_3_ exchange activity in normal rabbits (N = 4). (NU: normal untreated; IU: inflamed untreated; N + KT: normal treated with ketotifen, I + KT: inflamed treated with ketotifen).

Cl:HCO_3_ exchange, defined as HCO_3_-dependent and DIDS-sensitive ^36^Cl- uptake, was significantly reduced in villus cell BBM vesicles (BBMVs) from rabbits with chronic intestinal inflammation compared with the control. However, ketotifen treatment of rabbits with chronic enteritis resulted in reversal of inhibition of Cl:HCO_3_ exchange in villus cell BBMVs but had no effect in normal rabbits (836.7 ± 12.5 pmol/mg protein/min in normal; 240.5 ± 13.4 pmol/mg protein/min in inflamed; 880.9 ± 17.1 pmol/mg protein/min in normal + ketotifen; 893.2 ± 6.7 pmol/mg protein/min in inflamed + ketotifen; Figure 2). These results indicated that Cl:HCO_3_ downregulated during chronic intestinal inflammation was reversed by in vivo treatment with ketotifen, a mast cell stabilizer.

### 2.3. Kinetic Studies

**Figure 3:**
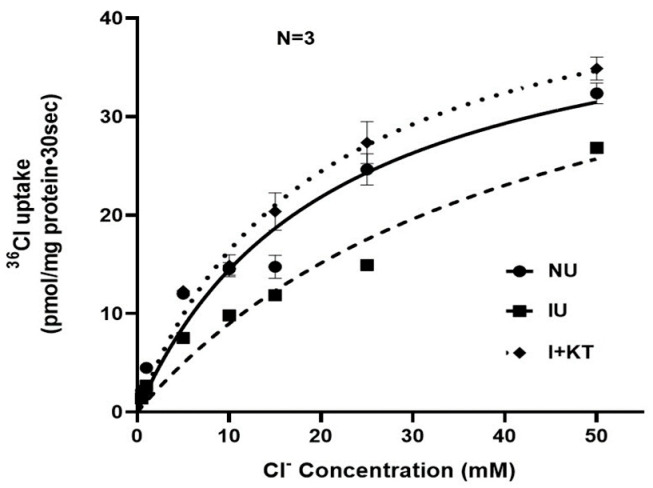
Kinetics study of Cl:HCO_3_ exchange in rabbit villus cell BBMVs. HCO_3_-dependent and DIDS-sensitive ^36^Cl uptake is shown as a function of varying concentrations of extra vesicular Cl at 30 secs. Uptake numbers derived from kinetics experiments were analyzed for Michaelis–Menten kinetics using non-linear regression data analysis to derive kinetic parameters *K_m_* and *V_max_* using GraphPad Prism 9 (NU: normal untreated; IU: inflamed untreated; I + KT: inflamed treated with ketotifen). Please note that some error bars are smaller than the symbols so are not visible in the graph.

Kinetics studies were performed to determine the mechanism of ketotifen-mediated reversal of inhibition of Cl:HCO_3_ exchange in the chronically inflamed intestine. Analysis of the uptake data demonstrated that as the concentration of extra-vesicular Cl was increased (Figure 3), ^36^Cl uptake was stimulated and subsequently became saturated under all experimental conditions. Moreover, *V_max_* for chloride uptake remained unchanged under all experimental conditions (*V_max_*: 44.6 ± 4.5 pmol/mg protein/30 s in normal, 48.2 ± 6.4 in inflamed, and 48.1 ± 3.5 in inflamed + ketotifen; n = 3). However, the affinity of Cl:HCO_3_ exchange activity was significantly reduced in the chronically inflamed rabbit intestine, but was reversed to normal levels by ketotifen treatment (*K_m_*: 20.8 ± 4.5 mM in normal, 43.7 ± 9.9 inflamed, and 19.4 ± 3.1 inflamed + ketotifen; n = 3, *p* < 0.01 normal vs. inflamed, *p* < 0.01 inflamed vs. inflamed + ketotifen). These data indicate that the mechanism of inhibition of Cl:HCO_3_ exchange reversal by ketotifen during chronic intestinal inflammation was secondary to a restoration of the affinity for Cl rather than altered transporter numbers.

### 2.4. Determination of mRNA Expression by RTQ-PCR

**Figure 4:**
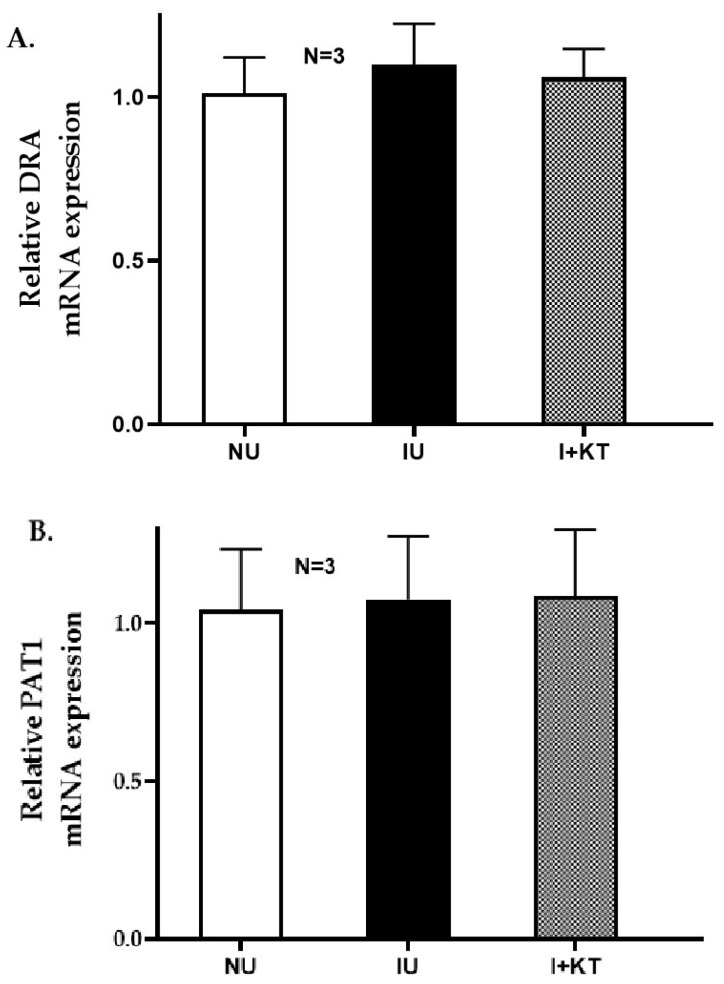
mRNA expression of rabbit ileal villus cell DRA and PAT1 determined by RTQ-PCR. DRA and PAT1 isoforms were responsible for Cl:HCO_3_ exchange activity in the rabbit ileal villus cells. RTQ-PCR studies showed that villus cells’ mRNA expressions of both (**A**) DRA and (**B**) PAT1 remained unaltered under all the experimental conditions in the rabbit model of chronic intestinal inflammation. (NU: normal untreated; IU: inflamed untreated; I + KT: inflamed treated with ketotifen).

To determine the molecular mechanism of mast cell-mediated regulation of the Cl:HCO_3_ exchangers DRA and PAT1, their mRNA expression in villus cells was determined by RTQ-PCR. The results showed that DRA and PAT1-specific mRNA expressions remained unchanged under all experimental conditions (Figure 4). This data was consistent with kinetic parameters that demonstrated that the mechanism of ketotifen-mediated reversal of inhibition of Cl:HCO_3_ exchanger activity in the villus cells from the chronically inflamed intestine was secondary to the restoration of the affinity of the exchangers for its substrate without a change in the exchanger numbers.

### 2.5. Western Blot Studies

**Figure 5:**
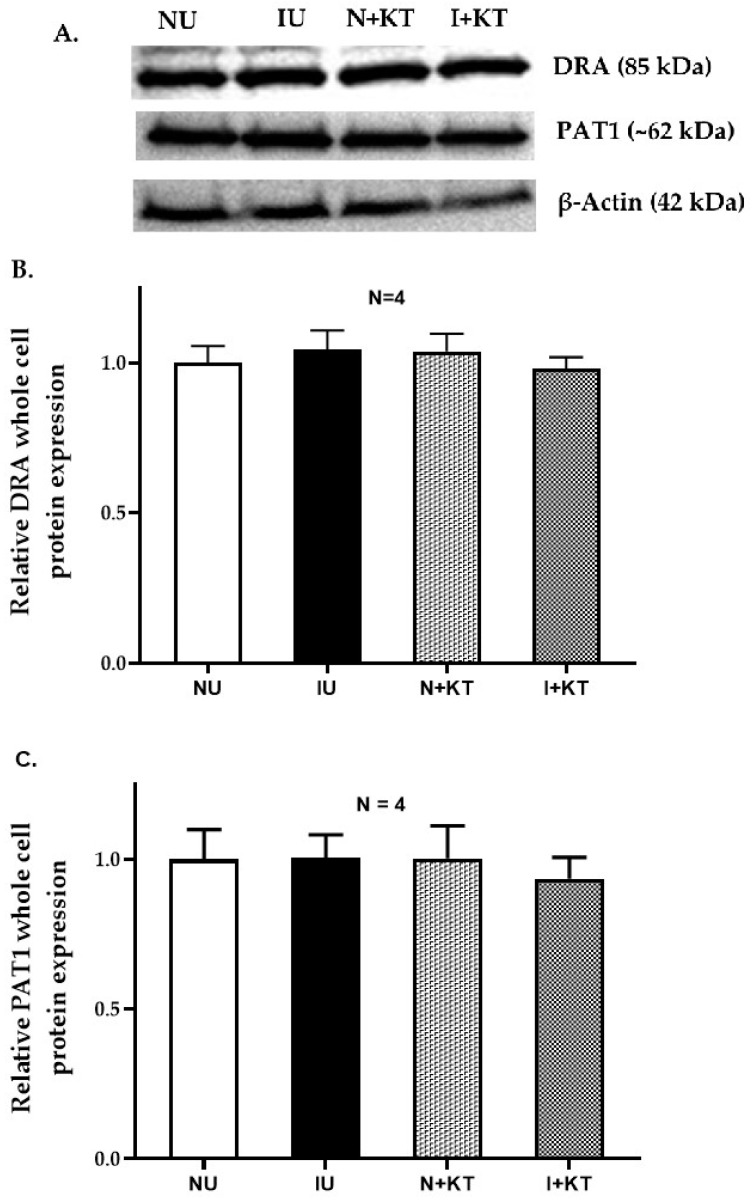
Western blot analysis for DRA and PAT1 immunoreactive protein in rabbit ileal villus cells. (**A**) The top panel is the representative image of the Western blot analysis. The whole cells’ DRA and PAT1 protein expression were unaffected in the chronically inflamed ileal villus cells as well as in ketotifen-treated rabbits with chronic enteritis. (**B**) Densitometric analysis of DRA protein expression. (**C**) Densitometric analysis of PAT1 protein expression. (NU: normal untreated; IU: inflamed untreated; N + KT: normal treated with ketotifen, I + KT: inflamed treated with ketotifen).

**Figure 6:**
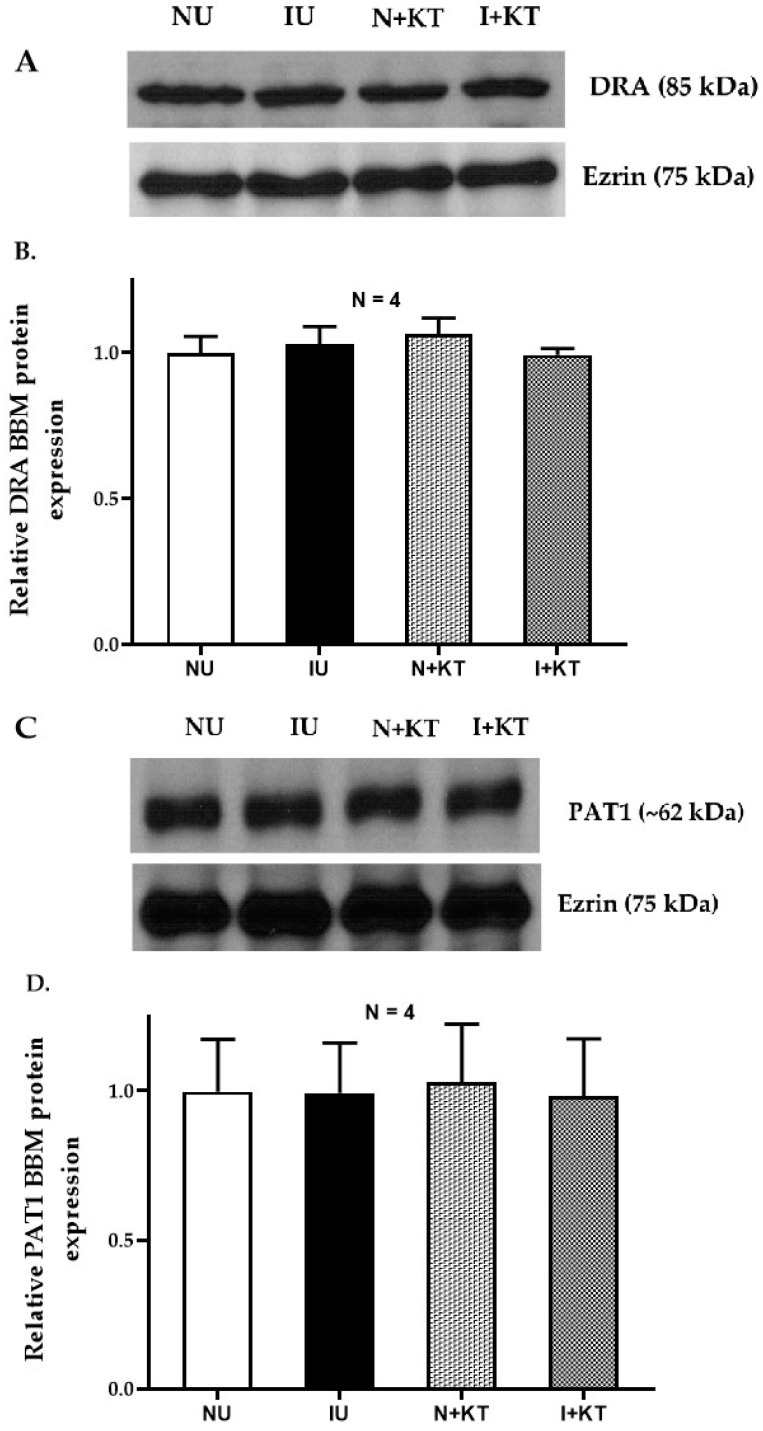
Western blot analysis for DRA and PAT1 protein in rabbit ileal villus cell BBM. (**A**,**C**) Representative blots showing BBM DRA and PAT1 protein levels in ileal villus obtained under the different experimental conditions. (**B**) Densitometry analysis of BBM DRA protein expression. (**D**) Densitometry analysis of BBM PAT1 protein expression (NU: normal untreated; IU: inflamed untreated; N + KT: normal treated with ketotifen, I + KT: inflamed treated with ketotifen).

DRA and PAT1 mRNA levels may not necessarily correlate with their functional protein expression in the BBM. Therefore, we determined their protein expression levels via Western blot analysis. There was no significant difference in the immunoreactive levels of DRA and PAT1 in the whole cells or in the BBM of villus cells obtained under the different experimental conditions. Densitometric quantitation confirmed these observations (Figure 5 and Figure 6). These data together with kinetic parameters and RTQ-PCR data suggest that the mechanism of inhibition of Cl:HCO_3_ exchanger activity by mast cells during chronic intestinal inflammation was secondary to a decrease in the affinity of the transporter for chloride without a change in the exchanger numbers.

## 3. Discussion

In this study, we used a rabbit model of chronic intestinal inflammation that had been previously characterized and established to be a suitable model to study mucosal changes, especially changes involving the absorption of nutrients and electrolytes in chronic intestinal inflammation. In this rabbit model, chronic ileitis was found to be associated with histopathology and mucosal injury similar to that seen in IBD, i.e., there was reduction in brush border enzymes and transport mechanisms, elevation in certain pro-inflammatory enzymes and chemotactic pro-inflammatory mediators, and alterations in oxidant/antioxidant mechanisms. Using this ideal model of chronic intestinal inflammation, the present study demonstrated that treatment of rabbits with chronic ileitis with mast cell degranulation inhibitor ketotifen did not significantly affect the morphology of the intestine but reversed the inhibition of Cl:HCO_3_ exchange. The mechanism of reversal of diminished Cl:HCO_3_ exchange by ketotifen in villus cells included restoration of the affinity of Cl:HCO_3_ exchangers without an effect on transporter numbers. The functional restoration of Cl:HCO_3_ exchange activity was further substantiated by molecular studies, where the mRNA expression and protein expressions of DRA and PAT1 (in whole cell and BBM) remained unaffected in the villus cells under all experimental conditions, indicating that the expression levels of DRA and PAT1 were not transcriptionally altered in inflammation or by ketotifen treatment. Thus, this study demonstrated that mast cell mediators probably mediated the inhibition of Cl:HCO_3_ exchange in villus cells during chronic intestinal inflammation.

It was previously demonstrated in a rabbit model that mast cells were responsible for the alteration of Na-dependent glutamine absorption in the small intestinal villus and crypt cells [18]. That specific study also demonstrated that the mast cell degranulation marker enzyme (β-hexosaminidase) levels were significantly increased in rabbits with chronic enteritis compared with normal rabbits. Most importantly, treatment of rabbits with chronic enteritis with ketotifen restored the β-hexosaminidase enzyme levels to normal in the small intestinal mucosa, confirming stabilization of mast cells by ketotifen. Thus, in the current study, the restoration of Cl:HCO_3_ exchange activity is also likely to have been due to the stabilization of mast cell degranulation via ketotifen treatment. In another study conducted in the same rabbit model of chronic intestinal inflammation, treatment with arachidonoyl trifluoromethyl ketone (ATMK), an inhibitor of arachidonic acid production, resulted in the restoration of chloride malabsorption mediated by DRA/PAT1. That study logically follows and substantiates the current study, since immunological activation of mucosal mast cells is known to cause the release of arachidonic acid from phospholipids in the cell membrane, becoming the substrate for various eicosanoids [6]. It should also be noted that mast cell mediator serotonin was found to regulate small intestinal chloride absorption [19] in normal rabbit intestinal enterocytes. Thus, the current study in conjunction with the previous studies confirms the role of mast cells as a key regulator of intestinal malabsorption of chloride during chronic enteritis.

Mucosal mast cells, in addition to their significant role in innate and adaptive immune responses, also play an integral role in maintaining gut homeostasis, host defense to external biological stimuli, and epithelial, endothelial, and neurological functions in the gut [20]. Several studies conducted in various animal models of IBD, as well as in human IBD, have demonstrated that increased mast cell proliferation and degranulation play a key role in the pathogenesis of IBD [4,6,21,22,23,24,25,26]. Moreover, it has also been suggested that the alterations in gut functions in the context of IBD can be the consequence of mast cell hyperreactivity due to dysbiosis of gut microbiota [27]. However, there is only limited documented evidence in literature describing the specific role of mast cells in the dysregulation of absorptive function of the gut in IBD [7,9,18,28,29,30,31]. Most of these studies were published more than two decades ago. Given the importance of mast cells in the pathogenesis of IBD, recent studies on understanding the role of mast cells in malabsorption of nutrients and electrolytes in IBD are clearly lacking. Although the current study and two other recent studies [27,28] have indeed indicated the role of mast cells in the regulation of nutrient and electrolyte malabsorption in the small intestine in IBD, none of these studies have demonstrated the specific role of mast cells in histopathological changes such as villus atrophy, crypt hypertrophy, infiltration of immune cells, ulcers, and fistulas, as well as fibroids and strictures such as those found in Crohn’s disease, though these changes are known to be associated with persistent mucosal inflammation. This is because of the fact that the rabbit model of chronic enteritis does not demonstrate full disease penetration when the histopathological changes are fully manifested, as in human Crohn’s disease, which is one of the limitations in using this animal model. Therefore, additional studies in humans or in other appropriate animal models are required to determine the role of mast cells in histopathological alterations associated with chronic intestinal inflammation and how targeting mast cells by long term treatment may affect these alterations.

DRA and PAT1 are the two predominant Cl:HCO_3_ exchangers involved in the absorption of chloride in the mammalian small intestine [32,33]. DRA has been previously implicated in chloride malabsorption and IBD-associated diarrhea [12,13,19,28,34,35]. Moreover, mutations in DRA have been shown to cause congenital chloride diarrhea, whereas mutated PAT1 has not been correlated with diarrheal phenotypes [32,36,37,38]. A study undertaken in the SAMP1 model of spontaneous chronic ileitis indicated that DRA rather than PAT1 was responsible for chloride malabsorption in the small intestine [39]. However, it is unclear whether dysregulation of PAT1 contributes to the malabsorption of chloride in the rabbit model of IBD. This study indicates that both DRA and PAT1 are both responsible for the dysregulation of chloride absorption in the rabbit model of chronic ileitis. It is of interest that in this and multiple other studies, the mechanism of coupled NaCl malabsorption resulting in diarrhea is secondary to an alteration in Cl:HCO_3_ but not Na:H exchange. Thus, better understanding of the regulation of DRA and PAT1 in the pathogenesis of diarrhea in chronic enteritis, as seen in IBD, is necessary to delineate better treatment modalities.

In conclusion, malabsorption of chloride was mediated by the activation and degranulation of mast cells in a rabbit model of chronic enteritis. Malabsorption of chloride was due to the functional downregulation of the villus brush border membrane Cl:HCO_3_ exchangers DRA and PAT1 secondary to altered affinity of the exchanger for Cl, which was restored to normal levels by the mast cell stabilizer ketotifen.

## 4. Materials and Methods

### 4.1. Rabbit Model of Chronic Enteritis

**Figure 7:**
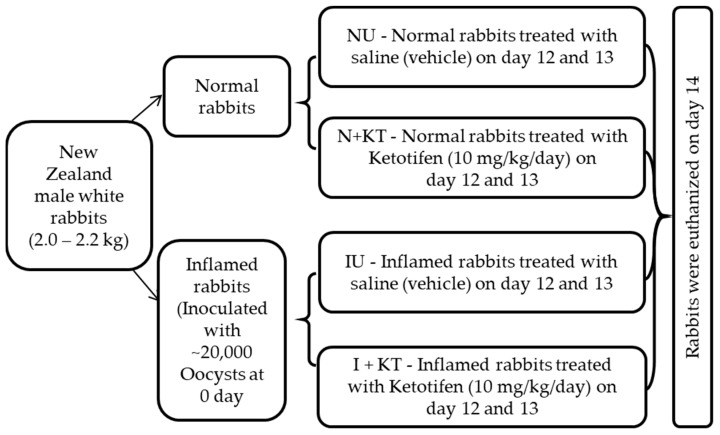
Flow chart demonstrating the experimental plan for rabbit inflammation and treatments.

New Zealand white rabbits (males) weighing 2.0–2.2 kg were purchased from Charles River Laboratories (PA, USA) and had a week of acclimatization before they were entered into the experimental protocol. They had free access to water and rabbit diet (5321-Laboratory Rabbit Diet; PMI nutrition International, St. Louis, MO, USA), and were housed individually in stainless steel cages under a regulated temperature of 22 ± 2 °C and humidity of 50–70% with controlled 12 h light-and-dark cycle. All the experimental procedures in this study were carried out in accordance with the ethical rules and regulations of Marshall University’s Institutional Animal Care Ethical Committee (Protocol Reference number 731, approval date 6 January 2020). After a week of acclimatization, rabbits were divided into four cohorts, each containing 4 animals (Figure 7). Animals in the 1st cohort were treated intramuscularly with saline (untreated control). Animals in the 2nd cohort were inoculated with *Eimeria magna* oocysts intragastrically as previously reported [15,40], to induce chronic intestinal inflammation. Rabbits in the 3rd cohort served as treatment control animals and were treated with the mast cell stabilizer ketotifen (10 mg/kg/day) for two days. Rabbits in 4th cohort included inflamed animals that were treated with ketotifen. All the treatments were performed on days 12 and 13 post-inoculation of oocysts or on corresponding days in the normal animals in the 3rd cohort. Animals were euthanized on day 14, as per Marshall University IACUC guidelines.

### 4.2. Histology Studies

A portion of the distal ileum was fixed in 10% (*v*/*v*) neutral-buffered formalin (Sigma Aldrich, St Louis, MO, USA) and embedded in paraffin. Sections (5 µm) from the formalin-fixed tissue were obtained with a microtome and were mounted on glass slides. Paraffin was removed from the sections by incubating the slides with xylene, and sections were hydrated gradually by incubating with graded ethanol. Sections were stained with hematoxylin and eosin (H&E; Sigma Aldrich, St Louis, MO, USA) staining.

### 4.3. Villus Cell Isolation, BBM Vesicle Preparation and Uptake Studies

To isolate small intestinal villus cells from the rabbit ileum, the ileal lumen was filled and incubated for 3 min with a cell isolation buffer (0.15 mM EDTA, 112 mM NaCl, 25 mM NaHCO_3_, 2.4 mM K_2_HPO_4_, 0.4 mM KH_2_PO_4_, 2.5 mM L-glutamine, 0.5 mM β-hydroxybutyrate, and 0.5 mM dithiothreitol; gassed with 95% O_2_, and 5% CO_2_, pH 7.4, at 37 °C) followed by gentle palpitation for another three minutes to facilitate villus cell isolation. The isolated cells suspended in the buffer were decanted from the lumen, separated by centrifugation (100 g for 3 min), and immediately flash frozen and stored at −80 °C. BBMVs (BBM vesicles) were prepared with the frozen isolated villus cells by CaCl_2_ precipitation and differential centrifugation technique. BBMVs were resuspended in an appropriate vesicle medium as described previously [16]. Then, ^36^Cl uptake experiments were performed on BBMVs to determine functional activities such as Cl:HCO_3_ uptake and kinetics using previously described protocols [16,28]. Uptake numbers derived from kinetics experiments were analyzed for Michaelis–Menten kinetics using a non-linear regression data analysis to derive kinetic parameters (GraphPad Prism 9.1.0; San Diego, CA, USA).

### 4.4. Real Time-Quantitative PCR (RTQ-PCR)

Total RNA was extracted from villus cells using an RNeasy mini kit (74106; Qiagen, Germantown, MD, USA) using the manufacturer’s protocol followed by first-strand cDNA synthesis with a High-capacity cDNA Reverse Transcription kit (4304437; Applied Biosystems, Foster City, CA, USA). Quantitative reverse transcription PCR experiments for rabbit β-actin (Oc03824857_g1), and rabbit-specific DRA (Oc03397193_g1) and PAT1 (Oc06726323_g1) were performed using TaqMan^®^ Gene Expression Assays obtained from Applied Biosystems. β-actin was used as an internal control to determine relative mRNA expressions of DRA and PAT1. RTQ-PCR was run for 40 cycles with each cycle set for 95 °C for 15 s and 60 °C for 1 min.

### 4.5. Western Blot Analysis

Western blot analysis of whole cell and BBM protein extracts was performed following standard protocols. Protein extracts were solubilized in RIPA buffer (50 mM Tris-HCl pH 7.4, 1% Igepal, 150 mM NaCl, 1 mM EDTA, 1 mM PMSF, 1 mM Na_3_VO_4_, 1 mM NaF) with protease inhibitor cocktail (SAFC Biosciences, Lenexa, KS, USA), mixed with sample loading buffer (100 mM Tris pH 6.8, 25% glycerol, 2% SDS, 0.01% bromophenol blue, 10% 2-ME), and separated on a custom-made 8% polyacrylamide gel. The separated proteins were transferred to BioTrace PVDF membrane and probed with anti-β-actin (SC-47778, Santa Cruz Biotechnology, Inc. Dallas, TX, USA), anti-DRA (SC-376187; Santa Cruz Biotechnology, Inc.), anti-PAT1 (SC-515230; Santa Cruz Biotechnology, Inc.) antibody raised in mouse [28,41], and Ezrin (MAB3822; EMD Millipore. Corp, Burlington, MA, USA) raised in mouse. The primary antibody bound to the target protein was detected with horseradish peroxidase conjugated Goat Anti-Mouse IgG (1706516; Bio-Rad Laboratories, 2000 Alfred Nobel Dr., Hercules, CA, USA) secondary antibody. The resulting chemiluminescence was measured by autoradiography with an ECL Detection Reagent (GE Healthcare, Chicago, IL, USA) and the specific protein densities were quantitated by Image J 1.53 t.

### 4.6. Protein Quantification

Protein quantitation for uptake experiments and for the Western blot studies were carried out using Lowry’s method with the DC^TM^ protein assay kit, according to the manufacturer’s protocol (Bio-Rad, Berkeley, CA, USA).

### 4.7. Statistical Analysis

All the results in this study are expressed as means ± SEM, calculated with GraphPad Prism 9.1.0 (San Diego, CA, USA) software. The “n” number indicates functional and molecular experiments performed with cells isolated from different animals. For all the uptake experiments, each “n” also indicates uptakes performed as a triplicate. Data generated were analyzed using two-way analysis of variance (ANOVA, Tukey’s multiple comparisons test) with GraphPad Prism 9.1.0 to derive statistical significance.

## Data Availability

For verifiable query, data will be provided to investigators actively engaged in intestinal transport physiology research.
